# GPCR Activation States Induced by Nanobodies and Mini-G Proteins Compared by NMR Spectroscopy

**DOI:** 10.3390/molecules25245984

**Published:** 2020-12-17

**Authors:** Philip Rößler, Daniel Mayer, Ching-Ju Tsai, Dmitry B. Veprintsev, Gebhard F. X. Schertler, Alvar D. Gossert

**Affiliations:** 1 Institute of Molecular Biology and Biophysics, ETH Zürich, 8093 Zürich, Switzerland; philip.roessler@mol.biol.ethz.ch; 2 Biomolecular NMR Spectroscopy Platform, ETH Zürich, 8093 Zürich, Switzerland; 3 Paul Scherrer Institute, Division of Biology and Chemistry (BIO), 5232 Villigen, Switzerland; damayer@ucsd.edu (D.M.); ching-ju.tsai@psi.ch (C.-J.T.); gebhard.schertler@psi.ch (G.F.X.S.); 4 Centre of Membrane Proteins and Receptors (COMPARE), University of Nottingham, Midlands NG7 2RD, UK; Dmitry.Veprintsev@nottingham.ac.uk; 5 Division of Physiology, Pharmacology & Neuroscience, School of Life Sciences, University of Nottingham, Nottingham NG7 2UH, UK

**Keywords:** GPCR, nanobody, mini-G, NMR

## Abstract

In this work, we examine methyl nuclear magnetic resonance (NMR) spectra of the methionine ε-[^13^CH_3_] labelled thermostabilized β_1_ adrenergic receptor from turkey in association with a variety of different effectors, including mini-G_s_ and nanobody 60 (Nb60), which have not been previously studied in complex with β_1_ adrenergic receptor (β_1_AR) by NMR. Complexes with pindolol and Nb60 induce highly similar inactive states of the receptor, closely resembling the resting state conformational ensemble. We show that, upon binding of mini-Gs or nanobody 80 (Nb80), large allosteric changes throughout the receptor take place. The conformation of tβ_1_AR stabilized by the native-like mini-G_s_ protein is highly similar to the conformation induced by the currently used surrogate Nb80. Interestingly, in both cases residual dynamics are present, which were not observed in the resting states. Finally, we reproduce a pharmaceutically relevant situation, where an antagonist abolishes the interaction of the receptor with the mini-G protein in a competitive manner, validating the functional integrity of our preparation. The presented system is therefore well suited for reproducing the individual steps of the activation cycle of a G protein-coupled receptor (GPCR) in vitro and serves as a basis for functional and pharmacological characterizations of more native-like systems in the future.

## 1. Introduction

G protein-coupled receptors (GPCRs) are the most abundant family of integral membrane proteins, comprising more than 800 members. They have an essential role for the cell as they are responsible for sensing a vast range of external stimuli and consequently allow cells to respond to their environment. These stimuli detected by GPCRs are very diverse and range from the detection of hormones and small molecule ligands to sensing physical parameters such as light. Being key players in cell signaling, GPCRs are of exceptional medical interest and have been linked to numerous diseases. In 2017, more than 30% of all FDA approved drugs acted on 108 unique GPCRs [1]. Considering that more than 50% of all nonolfactory GPCRs have not yet been studied for their clinical relevance [1], GPCRs are an intense focus of ongoing biological and pharmaceutical research.

**Figure 1:**
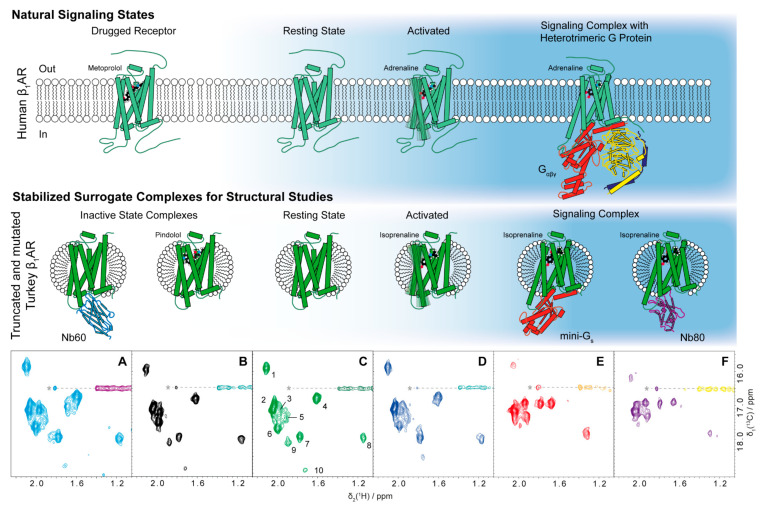
(Previous page): Overview of natural signaling states of β_1_ adrenergic receptor (β_1_AR) and their surrogate complexes used for structural studies. Top rows: simplified cartoons depicting the expected predominant conformations of the receptor in different complexes and activation states, with thick boxes indicating α-helices and thin boxes indicating β-strands. Top left: Antagonists such as the beta blocker metoprolol (ball and stick model) stabilize adrenergic receptors (pale green cartoon) in an inactive conformation, therefore abolishing signaling abilities by blocking access to the intracellular cavity. The same conformation is predominantly adopted by the resting receptor without any bound ligand. After binding of an agonist (adrenaline, ball and stick model) takes place, movements in helices 5 and 6 become possible (middle) and ultimately allow the binding of a heterotrimeric G protein (α subunit: red, β: yellow, γ: blue) to the receptor that accesses the intracellular cavity by insertion of the C-terminal helix of G_α_ and stabilizes the receptor in the active open conformation (right). Lower row, from left side: Surrogate complexes used for this study that resemble the natural signaling states of G protein-coupled receptors (GPCRs). All complexes are formed with a thermostabilized receptor (tβ_1_AR-TS, intense green cartoon) solubilized in a detergent micelle. Nanobody 60 (Nb60, cyan) and the antagonist pindolol (ball and stick model) can stabilize the inactive conformations. Activation of the receptor is induced by agonists such as isoprenaline. Complexes similar to the heterotrimeric G protein-bound GPCR can be obtained with nanobody 80 (Nb80, purple) or mini-G proteins (red). These cartoons are used throughout the manuscript to identify the intended complexes in the respective sample. [^13^C,^1^H]-NMR spectra of Metε-[^13^CH_3_] labelled tβ_1_AR of the corresponding surrogate complexes are shown below each complex (**A**–**F**, see figure captions and Materials section for details). The spectra are reproduced in later figures for more detailed comparisons. In (C), 10 of 11 expected signals are numbered. The dashed line with the asterisk denotes an artefact from an intense detergent signal outside of the shown spectral region. The blue background shading represents the degree of activation of the receptor, indicating stabilization towards the inactive state of the engineered tβ_1_AR-TS compared to the human wild-type receptor. Apo, agonist-bound and antagonist-bound proteins are based on the protein database (PDB) entry 2Y03. Homology of 6GDG and 6EG8 were used for the heterotrimeric G_αβγ_ complex. The cartoon for the mini-G_s_ complex was created in analogy to 6GDG. Complexes with Nb80 and Nb60 are based on 6H7J and 5JQH, respectively.

In general, GPCRs consist of seven transmembrane helices connected by six alternating intracellular and extracellular loops with the N- and C-termini in the extracellular and intracellular space, respectively (Figure 1, top row). The helices arrange in a barrel-like fashion and adopt different orientations towards each other depending on the activation state. In the case of the β_1_ adrenergic receptor (β_1_AR), binding of its agonists adrenaline or noradrenaline allows the rotation and outward movement of the intracellular end of transmembrane helix 6 (TM6) as well as smaller movements in TM5 and TM7. This change in structure opens an intracellular cavity that is used by heterotrimeric G proteins to bind to the GPCR by insertion of a C-terminal helix of the G_α_ subunit (Figure 1, top right). In the GPCR-bound state, the heterotrimeric G protein in turn gets activated by exchange of GDP for GTP in the α subunit and dissociates as G_α_ and G_βγ_ subunits to start the signaling cascade inside the cell. The desensitization of the receptor is mediated by GPCR kinases (GRKs), which phosphorylate the C-terminus and/or the intracellular loop 3 (ICL3) and consequently allow binding of a β-arrestin to the receptor (not shown in Figure 1). G protein signaling of the receptor is then stopped by either steric hindrance of the accessibility of the intracellular cavity or by promoting endocytosis of the receptor.

Drugs interact with GPCRs to alter the conformational equilibrium of the receptor and compete with natural ligands. Beta blockers are a prominent group of such drugs, including members such as metoprolol, which is among the most prescribed drugs in the US [2]. They act as antagonists for β adrenergic receptors, most notably for the β_1_AR present in the heart, and abolish the interaction with G proteins by keeping the receptor in the inactive conformation, ultimately causing a slower heart rate and reduced arterial blood pressure in patients.

### 1.1. Structural Biology of GPCRs

Structural biology has helped to gain deep insights into GPCR functioning by solving structures of receptors bound to diverse pharmaceutically active compounds and interacting proteins using X-ray crystallography and cryogenic electron microscopy (cryo-EM) [3,4,5]. Dynamic properties and equilibria between these states in solution and at physiological temperatures were studied by nuclear magnetic resonance (NMR) spectroscopy at atomic resolution [6,7,8,9,10]. Alternatively, electron paramagnetic resonance (EPR) spectroscopy was used to quantify the populations of protein in distinct conformations in frozen samples [11].

The named structural methods have in common that they pose stringent requirements to the sample, in terms of protein stability, amount and homogeneity. For pharmaceutical and biologically relevant insights, in principle the *wild-type* receptor should be studied in its *native* membrane environment. To date, none of the structural methods is able to derive information at the atomic level in situ and therefore surrogate systems are being studied. The most successful approach is to extract the receptor into a membrane mimicking phase such as detergent micelles or nanodiscs [12,13], and to screen for thermally stable variants of the target receptor, by exploiting sequences of different species and applying mutagenesis techniques. This led to several examples of long-term stable receptors which could be subjected to lengthy purification protocols in order to obtain homogenous preparations, which were suitable for crystallization, electron microscopy and NMR [3,14,15,16].

In this work, we focus on the human β_1_AR as an example, which has high clinical relevance for being targeted by the commonly prescribed beta blockers, but until now could not be directly structurally characterized. To bypass the intrinsic instability of the human receptor, surrogate receptors were designed. For the human β_1_AR, the homolog receptor from turkey (*Meleagris gallopavo*) was chosen as a base for the design of a thermo- and partially conformationally stabilized constructs since it was well characterized and previously shown to have high levels of expression at in insect cells [17,18]. The introduction of stabilizing mutations and the truncation of the termini and loops resulted in a stable tβ_1_AR-m23 construct, which was successfully used for structural studies [15,19,20]. (See Materials section for an exact description of the construct.) Just very recently the first crystal structure of a construct of human β_1_AR fused to a lysozyme and in complex with an active-state stabilizing nanobody or antagonist was published [21].

For structural studies of the complexes of GPCRs, it is also beneficial to replace the interaction partners of the receptor with surrogates since most interactors and their complexes are also intrinsically unstable. For this reason, for example, the agonist isoprenaline is commonly used as an alternative for the highly oxidation-sensitive adrenaline to activate tβ_1_AR. Furthermore, the heterotrimeric G protein is very difficult to produce in a stable form and cannot be readily used to form a characterizable complex with tβ_1_AR. Therefore, several surrogate strategies were explored, such as nanobodies and modified G_α_ subunits.

Nanobodies (Nb), the single chain camelid antibodies, have become a standard tool for the stabilization of GPCRs in different conformations [22]. Nb80 is a very prominent example for a G protein surrogate for the β adrenergic receptors. It was raised in llama by injection of agonist-bound β_2_ adrenergic receptor (β_2_AR) and was shown to increase the affinity of β_2_AR for the agonistic isoprenaline by the same factor as a G protein and furthermore allowed to solve the first supposedly active-like structure of a GPCR with a noncovalently bound agonist [23]. Nb80, and its affinity-maturated version Nb6B9 [24] thus became standard tools for the characterization of the active state [22,25,26] and standard surrogates for G proteins. Furthermore, nanobodies are a versatile tool and can also be used to stabilize other states. Nanobody 60 (Nb60), for example, binds preferentially to the inverse agonist-bound β_2_AR and was shown to stabilize an inactive state of the receptor [27,28].

In an alternative more natural approach to obtain a surrogate for the G protein-bound receptor, the complex of the heterotrimeric G protein was engineered into so-called mini-G proteins that comprise the minimal elements required for binding to a GPCR and to stabilize its active conformation [29,30,31]. Mini-G_s_ was the first protein of this series that was rationally designed from the GTPase domain of the G_s_ protein and validated by binding assays [29]. Today, multiple different mini-G protein constructs are available [31] and many GPCR structures were solved in complex with a mini-G protein [32,33] or the mini-G protein together with the βγ subunits of the heterotrimeric G protein [34,35], thereby increasing the understanding of the interaction between GPCRs and G proteins. While being a powerful tool in crystallography and cryo-EM, however, mini-G complexes have until now evaded the characterization by NMR spectroscopy.

In summary, X-ray crystallography and EM have allowed us to obtain snapshots of most of the relevant signaling states of a GPCR. These were obtained by selectively stabilizing individual states by the use of stabilizing mutations, truncation of loops and flexible termini, insertion of lysozyme, conformation-selective nanobodies and other binding partners such as G proteins or their mini-G variants.

With NMR, the dynamic properties of the individual states and the equilibria and transitions between them can be characterized, and therefore NMR studies have prominently accompanied the structural biology efforts and have led to several insights into the behavior of GPCRs [7,8,9].

### 1.2. Particular Challenges for NMR Studies on GPCRs

For NMR studies, GPCRs present some particular challenges. GPCRs are in a molecular weight range of 80–150 kDa for a detergent solubilized receptor in a micelle, which is above the size limit for conventional NMR techniques (25 kDa). Such large systems can be studied by combining deuterium incorporation and transverse relaxation optimized spectroscopy (TROSY) [36,37,38]. However, pharmaceutically relevant GPCRs often have to be produced in higher eukaryotic expression systems that do currently not allow for high levels of deuterium incorporation [39,40,41]. This leads to the necessity of long measurement times in the range of days for a sample at room temperature and thus only sufficiently stable samples can readily be studied. Rather stable GPCRs such as the human β_2_AR can be studied with only few stabilizing mutations [42,43]; however, most receptors tend to degrade quickly and therefore evade efforts to obtain spectra.

In contrast to X-ray crystallography and EM, where the receptor can be optimized and selected to represent a single state, a receptor that can move through its activation cycle is desired in order to study the conformational plasticity of the receptor and its interplay with a multitude of effectors by NMR. The thermo- and conformationally stabilized tβ_1_AR-m23, which is essentially locked in the inactive state, required the reintroduction of two crucial tyrosine residues (Y227^5.58^ and Y343^7.53^) in the receptor to restore its ability to activate G proteins [44]. This activation competent construct (further simply referred to as tβ_1_AR-TS for thermostabilized) and its derived versions represent suitable surrogates for the human receptor to study interactions with a diverse set of small molecule ligands and interacting proteins [44,45,46,47,48]. Using the stabilized tβ_1_AR and different ligands and nanobodies as interactors, the Grzesiek and Nietlispach groups have been able to characterize different aspects of this receptor [44,45,46,47,48].

### 1.3. Analysis and Interpretation of NMR Spectra

In this study, NMR spectroscopy was used as the primary analytical technique. 2D-[^13^C,^1^H]-correlation NMR spectra were recorded on receptor preparations, which have been produced with ^13^C-isotope labelled methyl groups of methionine (Figure 1A–F). For non-NMR experts, the information contained in such spectra shall be briefly introduced. The construct tβ_1_AR-TS contains 11 methionine residues (see figure (middle) for approximate location of the methionine residues on the receptor), and therefore the same number of individual signals is theoretically expected to be visible in these spectra—i.e., in absence of overlap or dynamic processes that can lead to disappearance of signals. A total of 10 signals can unambiguously be identified (Figure 1C); however, signals 2, 3, 5 and 6 are overlapped and it is therefore well possible that an additional signal is present in this region. In such spectra, the position of the signal on the two chemical shift axes reports on the chemical environment of the methyl group and the shape of the signal reflects its dynamics. The local chemical environment may change due to binding of another molecule in close proximity or due to allosteric changes that occur upon binding at a more distant site and this is reflected in a changed chemical shift. Without resonance assignments, direct and allosteric effects cannot, in principle, be distinguished. However, if only three methionine residues are in proximity of the intracellular cavity, and binding of an intracellular effector induces changes in essentially all signals, a large allosteric conformational change can be expected in order to explain the changing environment of distant sites. (e.g., see changes of the signals between Figure 1D,E, which indicate large conformational changes). Information about dynamics is contained in the shape of the signals. If a methyl group dynamically jumps between different chemical environments on a μs to ms timescale, then the signal is typically broadened. For example, signal 1 in Figure 1C is broadened in Figure 1E, indicating that this methionine residue is changing between environments on a μs to ms timescale. Another type of dynamics, very fast motions on the ps to ns timescale, will lead to sharp and intense signals. The sharp and intense signal 2 therefore most likely stems from the highly flexible N-terminal methionine.

Each resonance signal can be identified with a specific methyl group of the receptor. For the construct tβ_1_AR-TS we use here, however, no resonance assignments could be obtained up to now and published assignments from similar constructs [45] were not unambiguously transferrable. The difficulty in obtaining assignments for this construct is that the established NMR methods cannot be applied for large proteins produced in mammalian cells. Assignments were obtained in comparable cases [42,43,44,45] by mutagenesis of individual methionine residues. We thus prepared alanine mutants for all methionines. For most of these mutant proteins, a sample could be produced, but the resulting spectra were not unambiguously interpretable (Supplementary Figure S1). Therefore, instead of working with tentative assignments, we limited our interpretation in most cases to the analysis of the overall signal patterns and refrain from site specific assignments. As shown in the following, also in the absence of assignments, a wealth of information can be obtained and important conclusions can be drawn on the conformational similarity of states, allosteric effects and dynamic processes present in different effector-bound forms of the receptor.

In this work, we corroborate findings by the groups of Grzesiek and Nietlispach [44,45,46,47,48] using a single receptor preparation. We take advantage of the ample set of protein tools to form the surrogate complexes mimicking the relevant natural activation states of tβ_1_AR (Figure 1). Further, faster NMR techniques allow us to include the less stable mini-G_s_ protein in our studies and compare the results to those obtained with nanobodies. The highly similar signal shapes and positions for both complexes indicate a very similar behavior of the receptor in complex with mini-G_s_ and Nb80, and further strengthen the validity of previous results. Finally, competition experiments with antagonists further validate the functional state of our receptor preparation, which will allow us to reproduce pharmacologically relevant scenarios in the NMR tube and opens the way for quantitative studies of drug action. Taken together, these experiments represent steps towards ever more native-like preparations in the NMR tube to study relevant receptor functions at the atomic level.

## 2. Results and Discussion

The goal of this work was to reproduce the entire range of resting, inactive and active states of the β_1_AR receptor in an NMR tube in order to characterize the different states at atomic resolution (Figure 1, bottom row). The different states were induced using different effectors, namely low molecular weight ligands, nanobodies (Nb60 and Nb80) and the mini-G_s_ protein. Since all experiments were run on the same receptor construct, we were able to compare the effects of the different effectors.

In order to characterize the different signaling states of tβ_1_AR by NMR, we assembled the desired complexes using Metε-[^13^CH_3_] labelled tβ_1_AR-TS in *n-*decyl-β-d-maltopyranoside (DM) detergent micelles together with unlabeled interactor proteins and small molecule ligands. On these samples we recorded XL-ALSOFAST-[^13^C,^1^H]-HMQC experiments and compared the obtained NMR fingerprints (see figure captions and Methods section for details). The tβ_1_AR-TS construct used in this study contains 11 methionine residues (including the N-terminal methionine) and is therefore more natural than constructs from similar studies where natural methionine residues have been removed to increase the simplicity of spectra [45]. Furthermore, the ability of a stabilized tβ_1_AR construct with reintroduced Y227^5.58^ and Y343^7.53^ to activate G proteins was already established in previous studies [44]. For this reason, we will refer to the conformation adapted by the receptor in complex with mini-G_s_ or Nb80 as the active state, whereas the conformation induced by inactivating antagonists will be referred to as the inactive state. 

### 2.1. Inactive States of the Receptor Can Be Observed with Antagonists or Nb60

**Figure 2:**
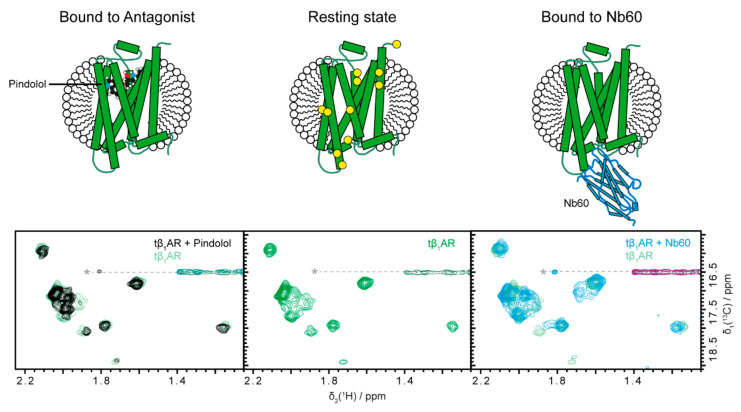
Stabilization of inactive states by drugs and Nb60. The inactive states of tβ_1_AR-TS can be stabilized by addition of the small molecule antagonist pindolol (ball and stick model, left) to the resting receptor (middle) or by addition of Nb60 (cyan, right). Only minor changes in chemical shift arise, indicating that the equilibrium of the resting receptor already strongly prefers the inactive state. Yellow circles indicate the position of methionine residues in the receptor. The dashed line with the asterisk denotes an artefact from an intense detergent signal outside of the shown spectral region. Methyl spectra were acquired at 25 °C on a 50 µM Metε-[^13^CH_3_] labelled tβ_1_AR-TS sample in 20 mM HEPES (pH 7.5), 300 mM NaCl and 0.2% *n*-decyl-β-d-maltopyranoside using the XL-ALSOFAST [^13^C,^1^H]-HMQC experiment on a 900 MHz Bruker Avance-III HD spectrometer. The sample with pindolol contained 100 µM pindolol, 1 mM isoprenaline, 4 mM ascorbate and a stochiometric equivalent of mini-G_s_ to the receptor (see Figure 5). The sample with Nb60 contained 1 mM isoprenaline, 4 mM ascorbate and 2 stochiometric equivalents of Nb60.

In a first step, we examined the inactivated state, which can be induced by antagonistic drugs or Nb60 [28]. Binding of an antagonist such as pindolol (Figure 2 left) to the receptor causes only small changes to the spectrum when compared to the apo protein. Overall, most signals are unaffected by the binding, indicating that the general conformation is not altered by pindolol. One signal that is strongly affected by addition of pindolol is most likely situated in the binding pocket and most likely reports on direct binding. In summary, the apo state of tβ_1_AR-TS is therefore highly similar to the pindolol-bound inactive state. This finding is in line with the stabilizing mutations that shift the equilibrium towards the inactive state and therefore no large changes in the spectrum are expected after the addition of the antagonistic ligand pindolol, which further stabilized the inactive conformation [15]. 

In a next step, we compared the effects of the small molecule pindolol on the receptor with the ones induced by Nb60. Nb60 was raised against β_2_AR, and it was shown to stabilize one of its inactive conformations by ^19^F NMR [28]. To our knowledge, we show here the first spectrum of β_1_AR in complex with Nb60. Compared to pindolol binding, Nb60 binds at the effector site on the opposite face of the receptor, and a larger binding surface is involved in the interaction. However, there are only three methionine residues in direct proximity of the intracellular cavity (Figure 2, middle), and therefore only those signals could potentially experience direct binding effects. Again, most of the signals remain at very similar positions compared to the apo state, indicating binding, but hardly any conformational change. It is therefore quite remarkable that the small molecule pindolol and Nb60, binding at opposite sides of the receptor, produce very similar spectra, as they both induce an inactive state.

Multiple NMR studies on β_1_AR and β_2_AR have previously reported on the presence of at least two inactive states of the receptor [46,48,49]. With the presented spectra, we cannot directly demonstrate the presence of multiple inactive states. However, one indication is the nonuniform broadening of signals, which indicates residual flexibility, highlights conformational exchange between different inactive states. Another indication for multiple states are the slight deviations between the spectra in the presence of pindolol and Nb60; these differences could be partially attributed to stabilization of similar but slightly different conformations of the receptor. As, at present, we do not have a resonance assignment for this tβ_1_AR-TS construct, we cannot distinguish direct from indirect binding effects.

### 2.2. Receptor Activation Can Be Followed by NMR Spectroscopy via Isoprenaline and Mini-Gs Binding

Receptor activation can be accomplished with several effectors. Here, we tested the effects of the agonist isoprenaline (an adrenaline analogue), a nanobody (Nb80) and an engineered G protein surrogate (mini-G_s_).

**Figure 3:**
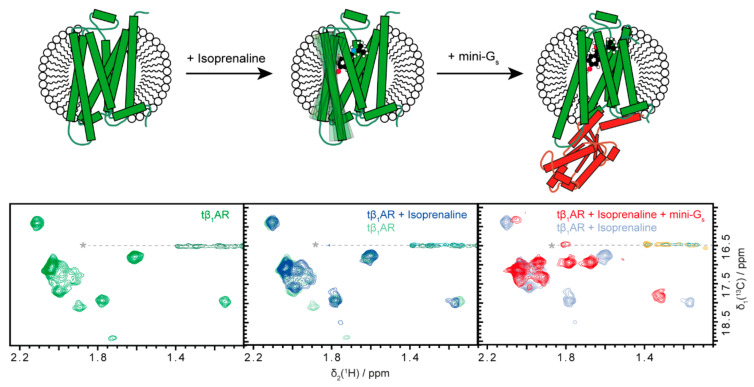
Crucial steps of the signaling pathway monitored by nuclear magnetic resonance (NMR). Shown is tβ_1_AR-TS in its resting state without any ligand (left), after addition of the agonist isoprenaline (ball and stick model, middle) and after addition of the G protein surrogate mini-G_s_ (red, right). Spectra of the respective states, recorded under the same conditions as in Figure 2, are shown below each cartoon. While agonist binding only leads to small changes in the spectrum, binding of mini-Gs induces lager conformational changes affecting the entire receptor. 1 mM isoprenaline and 4 mM ascorbate were added to obtain the agonist-bound state. The complex with mini-G_s_ was formed by addition of a stochiometric equivalent of mini-G_s_ to the agonist-bound receptor.

Compared to the spectrum of the apo protein, the spectrum of the agonist-bound receptor shows only small changes in chemical shifts of the methionine methyl signals, and weak broadening effects on two signals (Figure 3). Apparently, the dynamics of the receptor are slightly increased and the equilibrium between the active and inactive states is therefore only slightly shifted towards the active state in presence of the agonistic isoprenaline. This again reflects the intrinsic stabilization of this receptor construct towards the inactive state.

In contrast to the binding of an agonist, binding of the commonly used G protein surrogate, Nb80, causes large chemical shift perturbations (Figure 1F). All signals are shifted, even though there are only three methionines located in direct proximity of the intracellular cavity (Figure 2 middle), which could potentially sense direct binding effects. This clearly indicates an overall conformational change of the receptor, including allosteric effects towards the extracellular face of the receptor. Here, a comparison to Nb60 binding is also instructive: Nb60 binding was nearly silent on the receptor spectrum, as the conformation hardly changed (Figure 2, right). In contrast, binding of Nb80 with a similarly sized binding surface (Nb80: 1185 Å^2^, Nb60: 850 Å^2^, determined from 6H7J and 5JQH using epic [50]) results in a complete change of the spectrum. Therefore, given the long residence time of Nb80 and the overall even and rather sharp signal shapes in the Nb80-bound state, we infer that the equilibrium between the different states is strongly—if not completely—shifted towards the active conformation.

**Figure 4:**
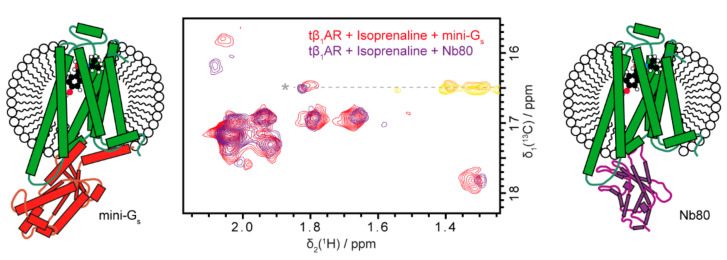
Nb80 and mini-G_s_ cause highly similar spectral changes after binding to tβ_1_AR. The obtained chemical shift patterns and signal shapes of the receptor are similar for the complexes formed with mini-G_s_ (red spectrum, left scheme) and Nb80 (purple, right), indicating the same induced active conformation for both G protein surrogates. The same conditions as in Figure 2 were used. The complex with Nb80 was formed by addition of two-fold stochiometric excess of the nanobody to the agonist-bound receptor.

NMR studies of the activated state of tβ_1_AR have so far exclusively been carried out using Nb80 or its affinity maturated analogue Nb6B9, instead of the more native-like G protein surrogate mini-G_s_, due to the limited stability of mini-G_s_ complexes. Using the newly developed XL-ALSOFAST-HMQC [51] experiment, we were able to obtain high quality spectra of mini-G_s_ complexes in a matter of hours. Therefore, we can compare the effects of mini-G_s_ and Nb80. Very similar large chemical shift perturbations are observable for the complex with mini-G_s_ as for Nb80 (Figure 4). The engineered G protein mimic is therefore also capable of shifting the equilibrium towards the active state.

It has been shown that mini-G and Nb80 binding increase the affinity of the receptor for agonistic ligands [23,29]. This occurs via allosteric effects throughout the receptor. In the NMR spectrum, the allosteric effects upon G protein binding are clearly visible, in that all resonances shift (except for the N-terminal methionine). This means that, by intracellular binding of the G protein to the receptor, methionine residues in proximity of the extracellular ligand binding cavity are affected, reporting on an allosteric conformational change of the binding pocket, which—following pharmacological assay data—increases the affinity to the agonist.

### 2.3. The Mini-Gs and Nb80-Bound Conformations of tβ_1_AR-TS Very Closely Resemble Each Other and Display Residual Local Dynamics

The chemical shift pattern of the methyl spectra for the tβ_1_AR-TS complexes with Nb80 and mini-G_s_ closely resemble each other. Most chemical shifts are identical with only minor differences for a few groups, which most likely arise from direct contact of a given methionine residue with the interacting protein. Additionally, the signal on the top left (signal 1 in Figure 1C at δ(^13^C) = 16 ppm) is strongly broadened compared to the apo state (Figure 1C) for both effectors, indicating residual flexibility for one part of the protein (Figure 4). This is in line with a recent ^19^F NMR study on a similar tβ_1_AR variant with Nb6B9, which could also show that the dynamics in TM7 are not fully arrested after formation of the active complex [46].

Our spectra suggest that the active state conformational ensemble as well as the dynamics of the tβ_1_AR-TS are the same for the complex with Nb80 and mini-G_s_. Moreover, the fact that both Nb80 and mini-G_s_ produce the same peak pattern suggests that the equilibrium is shifted completely to the active state.

The more natural-like mini-G_s_ have the same common binding interface to the GPCR as a heterotrimeric G protein and are therefore likely to induce a natural active conformation of the receptor. The fact that Nb80 induces the same conformation in tβ_1_AR-TS is therefore a valuable validation for the nanobody as a suitable tool to study active states of GPCRs. 

### 2.4. Pindolol Can Outcompete Isoprenaline and Fully Abolish the Interaction with Mini-G_s_

**Figure 5:**
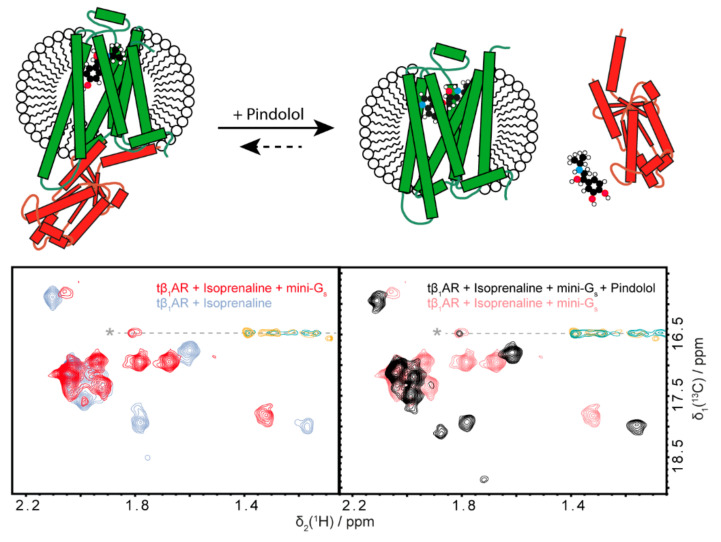
Pindolol can inhibit the interaction between tβ_1_AR-TS and mini-G_s_ in a competitive manner. The interaction between tβ_1_AR and mini-G_s_ (left) can be abolished by addition of the antagonist pindolol (right). The addition of pindolol causes the receptor to adopt an inactive conformation, further validating the functional state of the present protein preparation. The same conditions as in Figure 2 were used with 1 mM isoprenaline, 4 mM ascorbate and a stochiometric equivalent of mini-G_s_ present for the G protein surrogate complex. The pindolol-bound spectrum was obtained after addition of 100 µM pindolol to the previously mentioned sample.

A pharmacologically relevant setting can now be approximated with the use of mini-G_s_. The activated receptor complex consisting of adrenaline-bound β_1_AR and a G protein (Figure 1) is represented by the surrogates tβ_1_AR-TS, isoprenaline and mini-G_s_. The fully activated conformation of the receptor is stabilized by the two binding partners mini-G_s_ and isoprenaline and an antagonistic binder needs to displace both of them and compensate for this considerable binding energy. As evident from Figure 5, the antagonist pindolol can displace isoprenaline in the ligand binding pocket of the receptor and force it to adapt an inactive conformation. This ultimately leads to the complete abolishment of the interaction between tβ_1_AR-TS and mini-G_s_. Therefore, pindolol acts on the GPCR as is expected for a beta blocker.

## 3. Conclusions

This corroborative study illustrates how the dynamic signaling processes in GPCRs can be studied at the atomic level by NMR spectroscopy. Even in the absence of resonance assignments several important aspects of the GPCR conformational cycle under influence of a variety of small molecule and protein effectors could be studied. In summary, this study further corroborates the notion that resting states and inhibited conformations are highly similar as reflected in the respective NMR spectra, and that agonist binding only slightly shifts the equilibrium towards the G protein-bound conformation. Only upon actual binding of the intracellular effector, here mini-G_s_, the equilibrium is strongly shifted towards the active conformation, witnessed by an overall conformational change throughout the receptor. The latter conformations can be induced by two effectors, Nb80 and mini-G_s_, in conjunction with a small molecule agonist. The very similar NMR spectral fingerprints induced by Nb80 and mini-G_s_ further validate the use of the tool Nb80 as a G protein surrogate. Further, residual dynamics in the active state strengthen the notion of presence of several active subconformations [46]. It would be desirable to obtain resonance assignments in order to localize the dynamic processes in the G protein-bound state. Previous work by the Nietlispach group indicates that these dynamics mainly affect TM7 [46].

To date, the surrogate systems consisting of stabilized turkey β_1_AR and engineered binding partners have helped acquire important understandings of receptor functions and several details seen in the presented spectra hold promise of additional insights into the activation processes in GPCRs. In turn, desensitization processes on GPCRs have not yet been studied in as much detail as have activation processes. It will therefore be important to extend the NMR studies towards receptor desensitization to address, for example, biased agonism [52,53,54].

In the realm of pharmacology, however, the surrogate systems have limited relevance. Here, we have shown that pharmacologically relevant competition assays are possible in an NMR tube. However, for obtaining insights that are transferrable to human health and disease, it is necessary to extend such NMR studies to systems that are more closely related to the native human one. We hope that our contribution in producing isotope labelled receptor in human cell lines exemplified here will enable NMR studies of the human β_1_ receptor in the future. Encouraging results towards structural studies of systems that resemble ever more closely the natural ones are constantly emerging [21,55]. We therefore trust that this study is yet another step towards unravelling the entire conformational landscape of native human receptors at the atomic level.

## 4. Materials and Methods

If not stated otherwise, all materials were obtained from Sigma-Aldrich (now Merck).

### 4.1. Construct, Expression and Purification of tβ_1_AR

A thermostabilized mutant of the β_1_ adrenergic receptor from *Meleagris gallopavo* based on previously identified modifications [15,56] was used in this work. Compared to the wild-type receptor (UniProt ID: P07700), the stabilized construct contains truncations at the N-terminus (Δ1–32), ICL3 (Δ244–271) and C-terminus (Δ369–483). The mutations R68S^1.59^, M90V^2.53^, C116L^3.27^, I129V^3.40^, D200E^ECL2^, A282L^6.27^, D322K^7.32^, F327A^7.37^, F338M^7.48^ and C358A^8.59^ were present (numbers in superscript indicate sequence positions in the Ballesteros–Weinstein system [57]), while Y227^5.58^ and Y343^7.53^ [44], which were mutated for most crystallization constructs, were kept. The construct was expressed as a fusion with a mEGFP containing Strep- and 1D4-tag according to the following sequence, where the bold fraction indicates the tβ_1_AR sequence.

> tβ_1_AR-mEGFP-2xStrep-1D4

MG**AELLSQQWEAGMSLLMALVVLLIVAGNVLVIAAIGSTQRLQTLTNLFITSLACADLVV**

**GLLVVPFGATLVVRGTWLWGSFLCELWTSLDVLCVTASVETLCVIAIDRYLAITSPFRYQ**

**SLMTRARAKVIICTVWAISALVSFLPIMMHWWRDEDPQALKCYQDPGCCEFVTNRAYAIA**

**SSIISFYIPLLIMIFVYLRVYREAKEQIRKIDRASKRKTSRVMLMREHKALKTLGIIMGV**

**FTLCWLPFFLVNIVNVFNRDLVPKWLFVAFNWLGYANSAMNPIIYCRSPDFRKAFKRLLA**

**FPRKADRRLH**GSGLEVLFQGPAAAMVSKGEELFTGVVPILVELDGDVNGHKFSVSGEGEG

DATYGKLTLKFICTTGKLPVPWPTLVTTLTYGVQCFSRYPDHMKQHDFFKSAMPEGYVQE

RTIFFKDDGNYKTRAEVKFEGDTLVNRIELKGIDFKEDGNILGHKLEYNYNSHNVYIMAD

KQKNGIKVNFKIRHNIEDGSVQLADHYQQNTPIGDGPVLLPDNHYLSTQSKLSKDPNEKR

DHMVLLEFVTAAGITLGMDELYKAAGSAWSHPQFEKGGGSGGGSGGSAWSHPQFEKGSGG

SEDLTETSQVAPA

The protein was produced in a stable tetracycline inducible HEK293S GnTi^−^ cell line, which was cultivated in suspension at 37 °C and 5% CO_2_ using V3 medium (Bioconcept). Protein production was started by addition of 2 μg/mL tetracycline (AppliChem) once cell concentrations reached 10^6^ cells/mL and the medium was afterwards supplemented with 5 mM sodium butyrate and 500 mg/l l-methionine-(methyl-^13^C). The cells were grown for 2.5 days and afterwards harvested by centrifugation for 5 min (100 × *g*).

Cells were resuspended in solubilization buffer (20 mM HEPES (pH 7.5), 300 mM NaCl, 1 mM EDTA, 10% glycerol, 1 mM PMSF and 2% *n*-decyl-β-d-maltopyranoside (*w*/*v*, Anatrace)) and shortly treated with a TURRAX IKA T18 disperser equipped with a S18N-19G dispersing tool. The lysate was stirred at 4 °C for 1 h to complete solubilization and was subsequently cleared by ultracentrifugation at 185,000 × *g* for 1 h. The clear supernatant was filtered using a 0.45 μm nitrocellulose membrane (Merck, MF-Millipore) and loaded onto a 5 mL StrepTrap HP column (GE). The column was washed with washing buffer (20 mM HEPES (pH 7.5), 300 mM NaCl and 0.2% *n*-decyl-β-d-maltopyranoside (*w*/*v*)) and protein was eluted in elution buffer (20 mM HEPES (pH 7.5), 300 mM NaCl, 2.5 mM desthiobiotin and 0.2% *n*-decyl-β-d-maltopyranoside (*w*/*v*)). 3C HRV protease (home-made) and 1 mM DTT were added to the eluate and incubated at 4 °C overnight to remove tags. The protein was concentrated using 50 kDa Amicon-Ultra concentrators (Merck Millipore) and a monodisperse fraction was obtained by size exclusion chromatography on a Superdex 200 column (GE) in washing buffer (see above). The purified protein was finally concentrated to 50 μM concentration for NMR experiments.

### 4.2. Purification of Nb80 and Nb60

Sequences of Nb80 and Nb60 were obtained from the PDB entries 6H7J [58] and 5JQH [28], respectively. The export signal sequence of pelB was added at the N-terminus in order to facilitate export into the periplasm of *E. coli*.

>Nb80

MKYLLPTAAAGLLLLAAQPAMASQVQLQESGGGLVQAGGSLRLSCAASGSIFSINTMGWY

RQAPGKQRELVAAIHSGGSTNYANSVKGRFTISRDNAANTVYLQMNSLKPEDTAVYYCNV

KDYGAVLYEYDYWGQGTQVTVSSGSGSHHHHHH

 

>Nb60

MKYLLPTAAAGLLLLAAQPAMASQVQLQESGGGLVQAGGSLRLSCAASGSIFSLNDMGWY

RQAPGKLRELVAAITSGGSTKYADSVKGRFTISRDNAKNTVYLQMNSLKAEDTAVYYCNA

KVAGTFSIYDYWGQGTQVTVSSGSGSHHHHHH

Plasmid DNA coding for Nb80 or Nb60 (pET28b-based) was transformed into *E. coli* BL21(DE3) Codon Plus RIL (Agilent). Cells were grown overnight at 37 °C on an agar plate supplemented with chloramphenicol and kanamycin. Some colonies were picked and transferred into a 50 mL Luria Bertani (LB) medium in an Erlenmeyer flask and this preculture was incubated overnight at 37 °C and shaken at 180 rpm. For expression at the liter scale, the preculture was diluted to an OD_600_ of 0.1 with fresh LB medium and the culture was shaken and grown at 37 °C until the OD_600_ reached 0.6 at which point the temperature of the shaker was set to 18 °C and half an hour later, 1 mM isopropyl β-d-1-thiogalactopyranoside (IPTG) was added. Cells were harvested the next day by centrifugation for 10 min at 6000 × *g* at 4 °C.

The cells were lysed in lysis buffer (50 mM TRIS (pH 8.0), 300 mM NaCl, 5 mM imidazole, 1 mM PMSF and 10% glycerol) with an LM10 Microfluidizer (Microfluidics) and the obtained lysate was centrifuged for 1 h at 35,000 × *g* at 4 °C. The supernatant was filtered with a 0.45 µm syringe filter (Sarstedt) and loaded onto a 5 mL Ni-NTA Superflow-Cartridge (Qiagen). The column was washed with washing buffer (50 mM TRIS (pH 8.0), 300 mM NaCl, 5 mM imidazole, 10% glycerol) and the protein was eluted with a gradient of elution buffer (50 mM TRIS (pH 8.0), 300 mM NaCl, 250 mM imidazole and 10% glycerol). Eluted protein was concentrated using a 10 kDa concentrator (Merck Millipore) and further purified by size-exclusion chromatography (SEC) on a Superdex-75 column in SEC buffer (20 mM HEPES (pH 7.5) and 300 mM NaCl).

### 4.3. Purification of Mini-G_s_

The mini-G_s_ protein was expressed with the following sequence.

>mini-Gs

MGHHHHHHENLYFQGIEKQLQKDKQVYRATHRLLLLGADNSGKSTIVKQMRILHGGSGGS

GGTSGIFETKFQVDKVNFHMFDVGGQRDERRKWIQCFNDVTAIIFVVDSSDYNRLQEALN

DFKSIWNNRWLRTISVILFLNKQDLLAEKVLAGKSKIEDYFPEFARYTTPEDATPEPGED

PRVTRAKYFIRDEFLRISTASGDGRHYCYPHFTCAVDTENARRIFNDCRDIIQRMHLRQY

ELL

The mini-G_s_ protein (pET15b based plasmid) was expressed analogously as Nb60 and Nb80. For purification, cells were lysed in lysis buffer (50 mM TRIS (pH 8.0), 300 mM NaCl, 5 mM imidazole, 1 mM PMSF and 10% glycerol) with an LM10 Microfluidizer (Microfluidics) and the obtained lysate was centrifuged for 1 h at 35,000 × *g* at 4 °C. The supernatant was filtered with a 0.45 µm syringe filter (Sarstedt) and loaded onto a 5 mL Ni-NTA Superflow-Cartridge (Qiagen). The column was washed with washing buffer (50 mM TRIS (pH 8.0), 300 mM NaCl, 5 mM imidazole, 10% glycerol) and the protein was step eluted with elution buffer (50 mM TRIS (pH 8.0), 300 mM NaCl, 250 mM imidazole and 10% glycerol). The buffer of the eluate was changed back to washing buffer using a HiPrep 26/10 Desalting Column (GE). TEV protease and 1 mM DTT were added and a second Ni-IMAC purification step was performed after overnight incubation to remove TEV protease and cleaved tag. The resulting flow-through was concentrated in a 10 kDa concentrator (Merck Millipore, Darmstadt, Germany) and the protein was further purified by size-exclusion chromatography on a Superdex-75 column in SEC buffer (20 mM HEPES (pH 7.5) and 300 mM NaCl).

### 4.4. NMR Spectroscopy

All spectra have been acquired on a 900 MHz (AV-III HD) spectrometer equipped with a TCI 1H-13C/15N-2H Cryoprobe (Bruker). All measurements were performed at 298 K in 3 mm sample tubes. Samples were concentrated to 50 µM tβ_1_AR in 20 mM HEPES (pH 7.5), 300 mM NaCl and 0.2% *n*-decyl-β-d-maltopyranoside (Anatrace). Isoprenaline was used in concentrations of 1 mM together with 4 mM ascorbate to counteract oxidation and the antagonist pindolol (Tocris Bioscience) was added at 100 µM concentration to samples. For the formation of G protein surrogate complexes, mini-G_s_ was used in stochiometric and nanobodies in two-fold stochiometric ratios to the receptor.

gs-XL-ALSOFAST-[^13^C,^1^H]-HMQC spectra with *τ*_1_ = 2.6 ms, *τ*_2_ = 1.4 ms, *d*_1_ = 500 ms, *t*_1_(^13^C) = 25 ms and *t*_2_(^1^H) = 53 ms have been recorded in order to obtain the methyl spectrum of the stabilized receptor.

## Supplementary Materials

The following are available online, Figure S1: Mutagenesis of methionine residues fails to provide unambiguous assignments for tβ_1_AR-TS.
