# Supplementary material for: GPCR Activation States Induced by Nanobodies and Mini-G Proteins Compared by NMR Spectroscopy
Source: Molecules. 2020 Dec 17;25(24):5984. doi: 10.3390/molecules25245984 (PMC7767065; doi:10.3390/molecules25245984)
Supplement: Supplementary file 1 [file molecules-25-05984-s001.pdf]

Supplementary Material

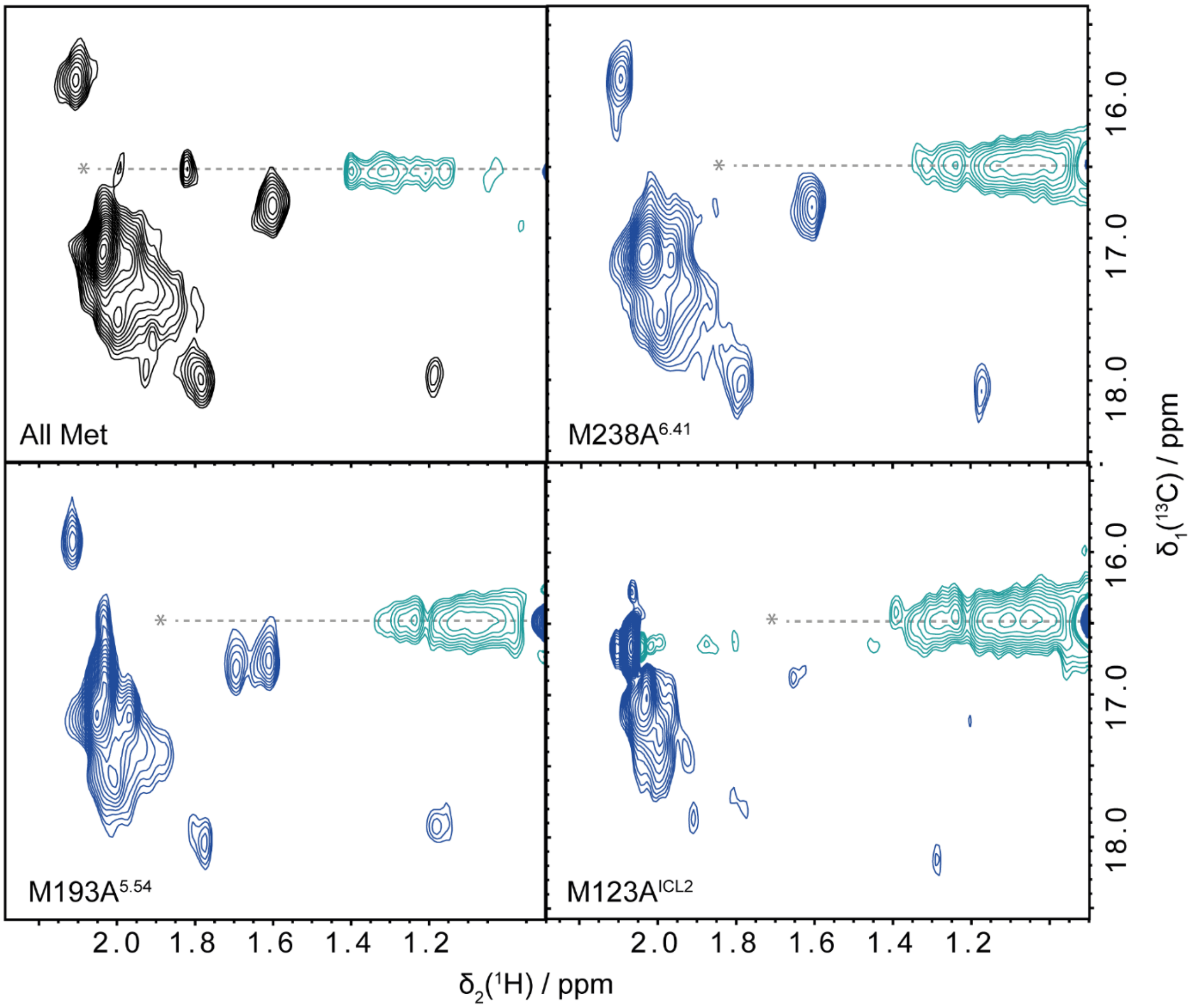

Supplementary Figure 1. Mutagenesis of methionine residues fails to provide unambiguous resonance assignments for tβ1AR-TS. 2D-[^{13}C,^{1}H] correlation spectra of Metε-[$^{13}CH_3$] labeled tβ1AR-TS (for exact sequence see *Materials and Methods*). The spectrum of the construct with all methionine residues present (All Met, top left) shows the familiar 10 signals (compare to e.g. Figure 1C in the main text). A single methionine-to-alanine mutation should lead to disappearance of a single signal in the spectrum. In practice however, purified mutants of this protein with single methionine-to-alanine mutations cannot be used for the assignment of the removed methionine residue since spectra show either no change (M238A), additional signals (M193A) or to many changes to unambiguously pin down the effect of the mutation (M123A). This inability to assign some positions of the receptor by mutagenesis most likely stems from the dynamic nature of the receptor by which mutations can stabilize conformations that would not be occupied in the unmutated receptor. All spectra have been acquired in the presence of 1 mM isoprenaline and 4 mM ascorbate. Spectral artefacts arising from a detergent signal are indicated by the dashed grey line and an asterisk. Numbers in superscript refer to the Ballesteros-Weinstein system for sequence numbering.
